# Characterisation of the genetic diversity of *Brucella *by multilocus sequencing

**DOI:** 10.1186/1471-2180-7-34

**Published:** 2007-04-20

**Authors:** Adrian M Whatmore, Lorraine L Perrett, Alastair P MacMillan

**Affiliations:** 1 Department of Statutory and Exotic Bacterial Diseases, Veterinary Laboratories Agency, Addlestone, Surrey, KT15 3NB, UK

## Abstract

**Background:**

*Brucella *species include economically important zoonotic pathogens that can infect a wide range of animals. There are currently six classically recognised species of *Brucella *although, as yet unnamed, isolates from various marine mammal species have been reported. In order to investigate genetic relationships within the group and identify potential diagnostic markers we have sequenced multiple genetic loci from a large sample of *Brucella *isolates representing the known diversity of the genus.

**Results:**

Nine discrete genomic loci corresponding to 4,396 bp of sequence were examined from 160 *Brucella *isolates. By assigning each distinct allele at a locus an arbitrary numerical designation the population was found to represent 27 distinct sequence types (STs). Diversity at each locus ranged from 1.03–2.45% while overall genetic diversity equated to 1.5%. Most loci examined represent housekeeping gene loci and, in all but one case, the ratio of non-synonymous to synonymous change was substantially <1. Analysis of linkage equilibrium between loci indicated a strongly clonal overall population structure. Concatenated sequence data were used to construct an unrooted neighbour-joining tree representing the relationships between STs. This shows that four previously characterized classical *Brucella *species, *B. abortus*, *B. melitensis*, *B. ovis *and *B. neotomae *correspond to well-separated clusters. With the exception of biovar 5, *B. suis *isolates cluster together, although they form a more diverse group than other classical species with a number of distinct STs corresponding to the remaining four biovars. *B. canis *isolates are located on the same branch very closely related to, but distinguishable from, *B. suis *biovar 3 and 4 isolates. Marine mammal isolates represent a distinct, though rather weakly supported, cluster within which individual STs display one of three clear host preferences.

**Conclusion:**

The sequence database provides a powerful dataset for addressing ongoing controversies in *Brucella *taxonomy and a tool for unambiguously placing atypical, phenotypically discordant or newly emerging *Brucella *isolates. Furthermore, by using the phylogenetic backbone described here, robust and rationally selected markers for use in diagnostic assay development can be identified.

## Background

Members of the genus *Brucella *are causative agents of brucellosis, a widespread disease of various animal species, and a common zoonotic infection of man [1]. While some countries have eliminated or substantially reduced the disease by extensive eradication programs it remains endemic in many areas of the world [2]. There is thus a substantial economic burden of brucellosis reflecting, either the costs of attaining and maintaining disease free status, or the cost of disease in terms of loss of productivity and control costs. Over many years *Brucella *taxonomists developed a classification system that recognized six classical species based on subtle phenotypic and antigenic differences and differential host specificity. Thus traditionally *B. abortus *(bovine), *B. melitensis *(caprine and ovine), *B. ovis *(ovine), *B. canis *(canine), *B. suis *(porcine, rangiferine, leporine) and *B. neotomae *(rodent) are recognised. Some of the classical species are divided into biovars although the distinction of some of these biovars is based on very slight differences and can be difficult and somewhat subjective. Multiple biovars of *B. abortus*, *B. melitensis *and *B. suis *are recognized currently [3] although the status of some biovars, particularly those of *B. abortus*, remains unresolved.

The traditional view on *Brucella *taxonomy was challenged some time ago on the basis of the high level of genetic relatedness indicated by DNA hybridization experiments [4]. This genetic conservation has since been confirmed by a variety of approaches including multilocus enzyme electrophoresis (MLEE) [5] and 16S rRNA sequencing [6]. Reflecting this, comparison of single nucleotide polymorphisms (SNPs) present in three complete *Brucella *genome sequences (representing three distinct classical species) indicates mean diversity between genomes of around 0.22% [7]. It was proposed that only one species, *Brucella melitensis*, should be recognized in the genus *Brucella *[3]. However, reflecting practical considerations, this option has not found widespread support with most opting to retain the nomenspecies designations. Formal moves to reverse this decision were initiated recently [8]. In recent years it has become clear that *Brucella *isolates are more widely dispersed than originally thought with the identification of isolates in various marine mammal species [9-12]. These isolates appear distinct from those previously seen in terrestrial mammals and diversity within this group of isolates has been identified by a variety of approaches [13,14]. As a result of these findings it has been suggested that these isolates represent one or more new *Brucella *species [11,15]. However, in part reflecting the ongoing debate regarding *Brucella *nomenclature, the new species designations have not yet been validly published and currently have no standing in bacterial taxonomy.

The genetic conservation within *Brucella *has resulted in past difficulties in establishing the true relationships between some classical *Brucella *species and biovars and in identifying molecular markers for some groups. For example, *B. canis *has long been considered very closely related to *B. suis *on the basis of a number of approaches including chromosomal maps [16], *omp *profiling [17,18], MLEE [5], AFLP [19] and insertion sequence typing [20] and its status as a distinct species has been questioned. Similarly, studies using AFLP and MLEE have indicated that *B. suis *biovar 5 is distinct from other *B. suis *isolates [5,19] and thus it is not clear whether there is justification for including *B. suis *biovar 5 in a taxonomic group with *B. suis*. Indeed, the status of *B. suis *as a single species has been questioned in light of a broader host specificity and because, in contrast to other classical species, no species-specific markers for *B. suis *have been identified [21].

In recent years the sequencing of multiple genetic loci in bacteria, usually but not exclusively housekeeping genes, (multilocus sequence typing or MLST) has rapidly gained acceptance as a tool for the characterization of microbial populations. The approach has been applied widely to microbial typing and epidemiological studies at both local and global levels as well as generating data that is ideal for studies of population structure and phylogenetic relationships [22]. In light of the conserved nature of the *Brucella *genomes MLST is likely to be of little value for local epidemiological studies. Tools such as VNTR based typing [23-25], indexing variation at more rapidly evolving markers, are likely to be far more informative in such scenarios. However, the unambiguous and defined nature of sequence typing is ideal to address the overall genetic structure of the *Brucella *population and the development of such a tool will provide a firm foundation on which to address the outstanding taxonomic issues

The aim of this study was to determine the sequences of multiple genetic loci in order to examine the relationships between *Brucella *isolates representing a geographically, and temporally, diverse collection of 160 isolates belonging to all the currently recognized classical *Brucella *species and biovars. The availability of such an extensive and unambiguous dataset facilitates a robust assessment of relationships within and between the classical species and biovars. It will also identify polymorphisms that are of potential value as diagnostic markers and provide a preliminary validation of their distribution. Furthermore the data generated in this study will help address some of the issues raised by the ongoing debate on the taxonomy of the group and will provide a database against which to compare any new, emerging or atypical *Brucella *isolates.

## Results

### Choice of loci

We selected 9 distinct genomic fragments for characterization in this study. Seven of the nine selected loci represent classic housekeeping genes of the type conventionally used in MLST because accumulated changes occur slowly and are believed to be selectively neutral (Table 1). The remaining loci used are a fragment of *omp25*, encoding a 25 kDa outer membrane protein, included as a potentially more variable surface marker that might facilitate discrimination between closely related classical species and a fragment, labelled int-*hyp*, that is largely intergenic though it does include the extreme 5' of a hypothetical protein. As shown in Table 1 the loci chosen are scattered around the *Brucella *genomes and thus change at one locus should be independent of other loci.

### Diversity of housekeeping genes

In all 4,396 nucleotides spanning 9 loci were sequenced from 160 isolates. For each isolate the sequences obtained at each of the nine loci were compared with those of every other isolate and sequences were designated distinct alleles if they differed at one or more nucleotide sites. Table 2 shows the sequence characteristics compiled from all 160 strains. The number of alleles detected ranges from 5 in the case of int-*hyp *up to 10 in the case of *glk *and *omp25*. The number of polymorphic sites present ranges from 5 in *gyrB *(1.07%) and *trpE *(1.03%) up to 12 in *omp25 *(2.45%). The d_N_/d_S _ratio [d_S _= average frequency of synonymous substitutions per potential synonymous site; d_N _= average frequency of nonsynonymous substitutions per potential nonsynonymous site] was calculated to determine the degree of selection in the sequence population. As expected in evolutionarily conserved genes the d_N_/d_S _ratio is substantially <1 for all housekeeping genes except *glk *(1.671). The d_N_/d_S _ratio for *omp25 *is also substantially <1 (0.0161) while this calculation is not relevant in the case of int-*hyp *as much of the sequence is intergenic. The % GC content of the various loci ranges from 55.75% (int-*hyp*) up to 62.67% (*glk*) in comparison to the overall genomic GC content of approximately 57%.

### Genetic relatedness of isolates

**Figure 1:**
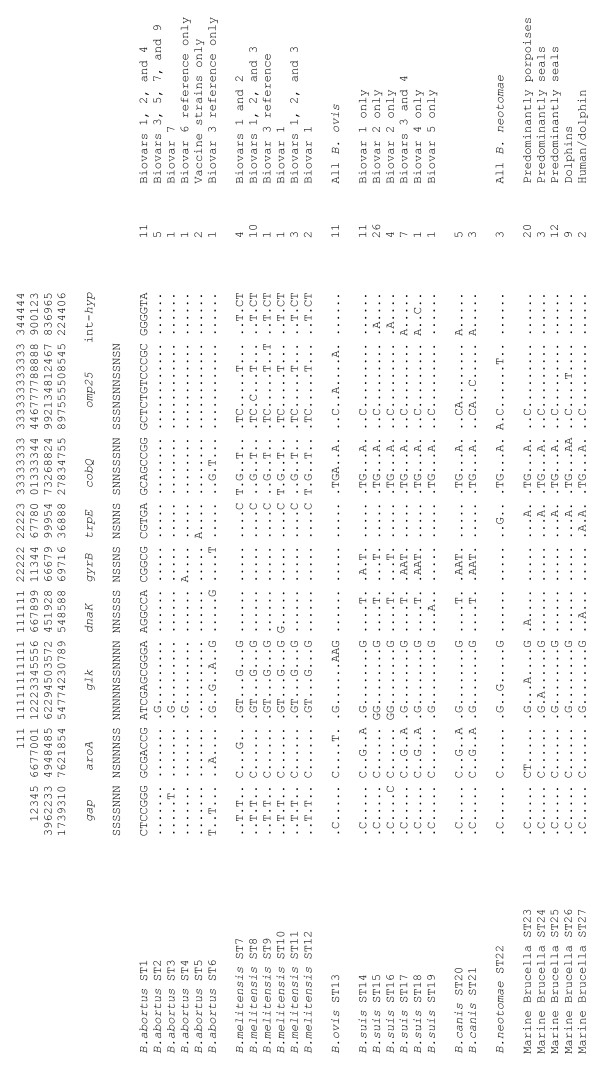
**Polymorphic sites detected at the nine loci examined in this study**. All polymorphic sites are shown relative to the ST1 sequence. Polymorphic sites are shown, while dots indicate nucleotides identical to ST1. The number of strains possessing each ST is shown at the end of each sequence string while the status of each site as a potential synonymous or non-synonymous change is shown by S or N respectively. The numbers above the gene designations represent the base number in the 4,396 bp concatenated sequence.

**Figure 2:**
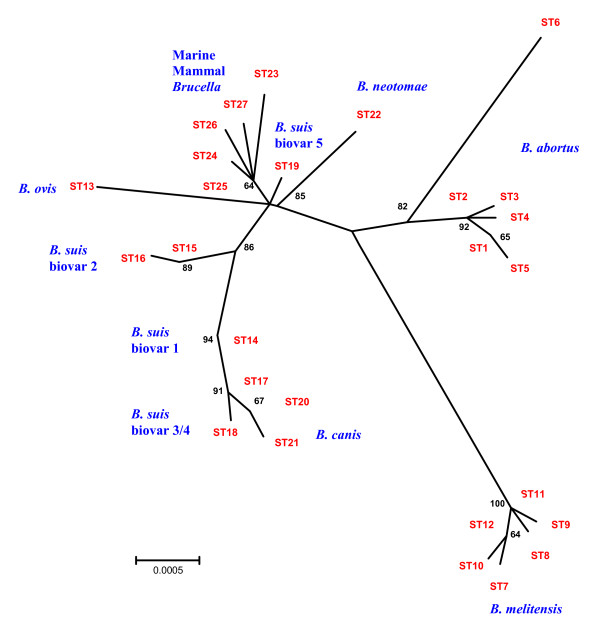
**Unrooted phylogenetic reconstruction of the relationships between STs**. This tree was constructed with the concatenated sequence data of the nine loci (4,396 bp) using the neighbour joining approach. The Jukes-Cantor model, which is based on the assumption that all nucleotide substitutions are equally likely, was used to determine genetic distances The percentage bootstrap confidence levels of internal branches were calculated from 1,000 resamplings of the original data.

Each distinct allele at each locus identified by sequencing was given an arbitrary numerical designation and each unique allelic pattern over all nine loci was identified as a sequence type or ST (Table 3). Overall 27 distinct STs were identified. The positions of all polymorphisms that relate to the 27 STs are shown in Fig. 1. The relationship between STs was examined by constructing a neighbour-joining tree from the concatenated nucleotide sequences of all 9 DNA fragments that comprised each ST (Fig. 2). Examination of the unrooted phylogenetic tree shows that STs fall into clusters that largely correspond to classical taxonomic divisions. *B. melitensis *and *B. abortus *both fall into well supported clusters although ST6 (corresponding to the *B. abortus *biovar 3 reference strain Tulya) is divergent from the remaining *B. abortus *STs. There is no obvious relationship between biovar and ST in the case of *B. melitensis*. There is some evidence of a possible relationship between biovars and ST in *B. abortus *but insufficient numbers of most biovars were examined to reach firm conclusions. Isolates of *B. neotomae *and *B. ovis*, both of which were found to represent a clone by this approach (i.e. a single ST) are both well separated from other groups. The 46 marine mammal isolates examined in this study fall into 5 STs that comprise a further cluster separated from terrestrial *Brucella *isolates although this separation is supported by a bootstrap value of only 64%.

It is immediately apparent from examination of the tree that the *B. suis *group appears rather genetically diverse in comparison with other classical species groups. Thus, there are 12 sites that are polymorphic within the *B. suis *group (Table 4). In contrast no polymorphisms were found in *B. neotomae *or *B. ovis*, 4 in the major *B. abortus *cluster (i.e. excluding ST6), 5 in *B. melitensis *and 8 in the marine mammal *Brucella*. However, all *B. suis *isolates other than the *B. suis *biovar 5 reference strain, (ST19), do comprise a distinct branch on the tree. In the case of *B. suis *STs are biovar specific with the exception of ST17 that consists of both biovar 3 and biovar 4 strains. There is clear separation of these biovar specific STs into strongly supported groups falling on this branch. Groups consist of two biovar 2 STs, a single biovar 1 ST and two biovar 3 and/or 4 STs. Comparison of the tree and its component sequence polymorphisms (Fig. 1) shows that biovars 3 and 4 are most closely related to biovar 1. Two *B. canis *specific STs are located at the terminus of this branch. These isolates differ from a *B. suis *biovar 3 and 4 ST (ST17) by only 1 or 2 polymorphic sites both located in *omp25 *(Fig. 1)

### Assessment of recombination

An assessment of the linkage between alleles from the different loci was performed in order to determine whether there is evidence for extensive recombination in the *Brucella *population (Table 5). Standardized I_A _(sI_A_) values were determined using the LIAN software program as this statistic is independent of the number of loci analysed in contrast to the originally described I_A _measure [26]. Standardized I_A _values are expected to be zero when a population is at linkage equilibrium (free recombination). Determination of sI_A _first involves computing the number of loci at which each pair of taxa differs. From the distribution of mismatch values a variance (V_o_) is calculated. This is compared with the variance expected for a population at linkage equilibrium (V_e_) in order to derive measures of sI_A_. LIAN also tests the null hypothesis of statistical independence of alleles (linkage equilibrium) at all loci by computer simulation. Input data is scrambled by resampling loci without replacement and computing a V_o _value for each resampled dataset. The significance of any difference between V_e _and V_o _is the frequency with which a V_o _value greater or equal to the original V_o _value is returned from the randomisation procedure. All analyses were carried out using both all isolates in a group and reduced to the level of STs (i.e. including only one isolate from each ST) to avoid potential bias due to a possible epidemic population structure.

When considering all 160 isolates the sI_A _was significantly different from zero both when including all isolates, (sI_A _= 0.2286 *P *= 0.001), and when analysis was reduced to the level of all STs (sI_A _= 0.1954 *P *= 0.001). This is consistent with evidence of strong linkage disequilibrium between loci and a clonal population structure with little or no recombination. Equally when the sI_A _was determined individually for all isolates of *B. abortus, B. melitensis, B. suis *and the marine mammal *Brucella *to test for evidence of recombination within the classical species there was no statistical evidence of recombination. However, the sI_A _was reduced to close to zero and the null hypothesis was supported poorly when considering STs alone in the case of *B. melitensis, B. suis *and the marine mammal *Brucella*. Though these values need to be treated cautiously as the number of STs in each group is very low this might suggest that there is some recombination within some of the classical *Brucella *species. The overall picture of a clonal population structure was supported by split decomposition analysis performed on a matrix of pairwise distances between the allelic profiles of all STs. This showed a radial distribution of strains with a tree-like structure (data not shown). The only evidence of network like structure, indicative of recombination, was seen in the *B. melitensis *cluster.

## Discussion

### *Brucella *genetic diversity

The data presented in this study represent a comprehensive study of genetic diversity within *Brucella *and the first application of multilocus sequencing to the group. Most of the data derive from housekeeping genes where genetic variation is considered largely neutral and thus such markers are considered to provide more reliable indications of genetic relatedness than genes subject to strong selection [27]. The availability of 4,396 bp of sequence data from each of 160 strains representing nine independent loci gives an unequalled resource with which to begin to understand the extent and nature of genetic diversity within the group. Furthermore, these data will further understanding of whether the traditional taxonomic designations of the group have a sound genetic basis and serve as a platform to assist and direct future taxonomic proposals. The use of multilocus sequence data has two particular advantages. Clearly, and of particular relevance in the case of a genetically conserved group such as *Brucella*, the additive use of multiple loci increases the discriminatory capacity compared to that that can be obtained when using a single target. Secondly, loci can be selected that are spaced far enough apart such that any pairs of alleles are unlikely to be inherited together by recombination. This is important as recombination can distort the apparent relationships between similar isolates if they are characterized at only a single locus. Thus studies based on multilocus approaches that buffer against possible recombination are more desirable than the characterisation of individual loci. It was recently suggested that such an approach should be applied by taxonomists to large samples of groups of closely-related bacteria, and especially to those where species delineation has historically been difficult, to determine whether genotypic clusters can be delineated, and to guide the definition of species [28].

Overall only 67/4396 nucleotide sites (1.5%) examined here are polymorphic equating to a variable site approximately every 66 bp. As expected, this is substantially more than the diversity detected based on the comparison of only three genomes [7] but still clearly indicates that the *Brucella *group is genetically rather uniform. This is in agreement with the recent observation based on genome sequences of a highly conserved genomic backbone within which species-specific DNA sequences and pseudogene distribution might correlate with different host preferences [29]. Within individual housekeeping gene loci diversity ranges from as little as 1.03% in *trpE *up to 2.32% in *glk *a level only marginally less than that seen in the outer membrane protein encoding fragment *omp25 *(2.45%). Nucleotide substitutions in genes coding for proteins can be either synonymous (do not change amino acid) or non-synonymous (change amino acid). Usually, most non-synonymous changes are expected to be eliminated by purifying selection, but under certain conditions Darwinian selection may lead to their retention. Therefore investigating the number of synonymous and non-synonymous substitutions provides information about the degree of selection operating on a system. As housekeeping genes are considered to undergo change that is selectively neutral or be subject to purifying selection the rate of synonymous change (d_S_) should be equal or greater than that of non-synonymous change (d_N_) giving a d_N_/d_S _ratio of <1. For 6 of the 7 housekeeping genes fragments examined in this study this is the case. However the ratio for *glk *is 1.671 suggesting that this gene fragment may be subject to positive Darwinian selection. Interestingly this fragment is by far the most variable of the housekeeping fragments and also has a GC content of 62.67% representing the fragment furthest removing from the genome average of 57%. The reasons for the evidence for selection operating on *glk *are unclear – it is possible that this gene may somehow influence pathogenic potential, transmissibility or tissue tropism. Alternatively this finding may reflect a hitchhiking effect where change at a locus subject to selection can drive change in neighbouring genes that are not themselves subject to selection [30]. The *omp25 *fragment encodes a surface marker potentially subject to selection. However, although it is the most variable fragment examined, most change in this gene appears synonymous and the d_N_/d_S _ratio is among the lowest apparent in this study.

The availability of multilocus sequence data enables an assessment of the extent of genetic recombination to be made by examining linkage equilibrium in a population. This not only gives an indication of the process of evolution in the group but also allows an assessment of whether meaningful phylogenetic interpretations can be made from multilocus sequence data. Analysis of linkage equilibrium in the complete population examined here was consistent with a clonal population structure with little or no recombination. This finding gives confidence in the utility of SNPs identified in this study as stable markers of particular phylogenetic groups. There is weak evidence that there may be some recombination within traditionally recognized species, notably *B. melitensis*, based on linkage equilibrium analysis and split decomposition analysis, but confirmation of this requires a much more extensive intraspecies study. These observations are consistent with the classical *Brucella *species evolving as isolated units in their preferred host species [21] where recombination may be theoretically possible but is restricted by ecological isolation.

### Implications for *Brucella *taxonomy

The traditional taxonomic designations of *Brucella *are in large part based on the apparent host specificity of the nomenspecies. As moves are ongoing to reverse the decision to define *Brucella *as a single species [8] and to formalize the taxonomic position of marine mammal *Brucella *the data described here will allow informed decisions to be to made that reflect genetic relationships as well as phenotypic properties and host associations. The definition of bacterial species is a subject of constant debate [31]. The gold standard approach is a 70% DNA-DNA hybridization cut-off though this method is infrequently used today. Most designations are based on 16S rRNA sequences though these are highly conserved with insufficient resolution to explore closely related populations [28]. Although the figure of <97% identity in 16S rRNA sequences is often quoted as a cut-off between species pragmatic definitions have led to situation where the extant genetic diversity in different species differs greatly [32]. Clearly, on the basis of both DNA-DNA hybridization and the 97% 16S rRNA sequence diversity cut-off, all *Brucella *could validly be considered members of a single species. However, equally the dendrogram constructed on the basis of concatenated sequence data (Fig. 2) does clearly separate most of the classically identified species on a genetic basis, albeit with low levels of diversity between clusters. Thus, there is clear separation of *B. abortus, B. melitensis, B. ovis*, and *B. neotomae *into well defined clusters or clones and therefore their identification as separate classical species does appear valid on both grounds of genetic separation and host specificity. The one possible exception here is the *B. abortus *biovar 3 reference strain Tulya (ST6) that branches off very early on the branch to all other *B. abortus *isolates. This isolate has previously been shown to be atypical relative to other *B. abortus *by VNTR analysis [24]. Interestingly, two distinct groups of *B. abortus *biovar 3 isolates were recently described [33], one of which corresponds to Tulya, while the other corresponds to local field isolates from Spain.

The situation with *B. suis *and *B. canis *is more complex. *B. suis *has long been considered to be a more diverse group of organisms than other classical *Brucella *species and this is confirmed by the branch lengths within the *B. suis *cluster seen here (Fig. 2). The status of *B. suis *biovar 5 as a *bona fide B. suis *isolate has been questioned. In support of this the reference strain does not cluster with other *B. suis *isolates on the basis of sequence data described here and appears more closely related to the marine mammal *Brucella*. The *B. suis *biovar 5 ST (ST19) has only two polymorphisms relative to the closest marine mammal ST (ST25) while there are five polymorphisms relative to the closest *B. suis *ST (ST15 – biovar 2). Isolates of the remaining four *B. suis *biovars do fall into a single branch. In contrast to *B. melitensis *there is clear separation of the distinct *B. suis *biovars (with the exception of biovars 3 and 4 that could not be separated by this approach). Thus, the phylogenetic tree generated here does support the classification of *B. suis *biovars 1–4 in a single species. There have been arguments for subdividing this group on the basis of distinct host specificities. This might be problematic from two aspects. Firstly the overlapping host specificities of *B. suis *biovars 1, 2, and 3, associated with pigs, makes separation on this basis difficult. Secondly, although there would be grounds on the basis of host specificity for separating *B. suis *biovar 4 which appears confined to rangifers, this biovar is genetically very closely related to *B. suis *biovar 1 being separated by only 2–3 polymorphisms. However, and conversely, if the same criteria were applied to *B. suis *biovar 4 as to *B. canis*, an argument could be made in favour of its classification as a separate species. *B. canis *has long been known to be closely related to *B. suis *[34] although its host specificity appears virtually absolute. This study confirms the close relationship of *B. canis *with *B. suis *biovar 3 and 4 isolates from which it differs at only 1 or 2 polymorphic sites both located in the *omp25 *fragment.

This study also allows us to address the ongoing debate surrounding the taxonomy of the recently discovered marine mammal *Brucella*. Following early observations that marine mammal strains varied both phenotypically and molecularly from other *Brucella *and within the 'group' [11,13,15,35-38] a number of controversial names that failed to follow the monospecific classification system were proposed. Initially a single marine mammal species, *B. maris*, was proposed [11]. Later, division into two species representing isolates originating from porpoises, dolphins and minke whales (*B. cetaceae*) and seals (*B. pinnipediae*) was proposed based on polymorphism at *omp2 *[15]. It was later acknowledged that a narrower host range may exist than that suggested by the *omp2 *locus [21]. In support of this genome profiling led to a suggestion that three distinct groups of marine mammal *Brucella *characteristic of dolphins, porpoises and seals should be recognized [39]. Our study confirms that the marine mammal *Brucella *do form a cluster distinct from all other species (Fig. 2) on the basis of the sequence data presented here. However, bootstrap support for this group is rather low and more data are required to confirm this clustering. While somewhat more diverse than *B. abortus *(excluding ST6) and *B. melitensis*, the marine mammal group has a similar level of 'intragroup' diversity as *B. suis *biovars 1–4. On this basis classification within a single species might be justified. However, this study also strongly supports the division into three groups with clearly distinct, though not absolute, host specificities (Table 3). Thus, in the extensive collection of marine mammal isolates sampled here, ST23 is strongly associated with porpoises (75% of isolates), ST26 is associated only with dolphins, and ST25, and its single locus variant ST24, are strongly associated with seals (80% of isolates). Although the sequence distances between these groups are small (e.g. ST 25 and ST26 differ by only two polymorphisms) if the criteria applied for *B. canis *speciation were applied here (i.e. speciation on the basis of distinct host specificity rather than substantial genetic separation) these groups could justifiably be classified as three distinct species. In this scenario the status of the remaining marine mammal ST, ST27, would remain unresolved. Although it appears genetically most closely related to ST25 the natural host of this ST is unclear. We found ST27 only twice, once in a bottlenose dolphin isolate [9], and once in a human infection where the source was not obvious [40].

### Intraspecies diversity and species-specific markers

The number of intraspecies polymorphisms detected and the presence of species-specific polymorphisms is shown in Table 4. Both *B. neotomae *and *B. ovis *represent a single clone in this study. The *B. neotomae *situation may simply reflect the paucity of available isolates representing this classical species but the *B. ovis *population used was more extensive and obtained globally. The lack of diversity in *B. ovis *relative to other classical *Brucella *species reflects recent findings by PFGE [41] and VNTR analysis [24]. As already discussed, if one excludes *B. abortus *Tulya, *B. suis *is the most diverse of the remaining classical species with biovars corresponding to STs. In contrast only a single polymorphism was detected within *B. canis *isolates. The remaining groups comprising *B. abortus*, *B. melitensis *and the marine mammal *Brucella *have between 4 and 8 'intraspecies' polymorphisms. There was no clear relationship between biovar and ST within *B. melitensis*. This finding is in agreement with observations based on multiple VNTR typing approaches where there appears not to be a strong correlation between genotype and biovar [24,25]. This suggests either that *B. melitensis *biovars do not correspond to true genetic groups, or that biotyping is so subjective that isolates are often incorrectly assigned masking any genetic relationship. *B. melitensis *biovars are notoriously difficult to distinguish as they are technically only serovars and thus their identification is particularly dependent on well trained staff and well-controlled preparation of monospecific sera. In contrast there is evidence that some *B. abortus *STs correspond to particular biovars but a much more extensive intraspecies study will be undertaken to fully assess this.

Species-specific markers in *Brucella *have sometimes been difficult to identify and for many years this hampered the development of molecular diagnostics. Recently Moreno *et al*. [21] noted that while the presence of various markers uncovered in recent years support the validity of host range as a criterion for defining *Brucella *species no species specific marker for *B. suis *in *omp *genes or elsewhere had been reported. As reported in Table 4 this study identified at least one apparently species specific SNP in all classical species except for *B. suis*. Thus, with further validation above and beyond the 160 strains examined in this study to confirm species-specificity, these SNPs represent potentially valuable diagnostic markers. While no species specific SNPs for *B. suis *were uncovered this in part reflects the fact that the classical species groupings are not entirely consistent with genetic groups. There are two SNPs that are specific to the major *B. suis*/*B.canis *genetic group (*dnaK*-1928; *gyrB*-2471). These SNPs are not present in *B. suis *biovar 5 reflecting the fact that this ST does not lie within the *B. suis*/*B canis *genetic group. In addition these SNPs are shared with *B. canis*, reflecting the position of *B. canis *as a terminal branch in the *B. suis *group. However, *B. canis *can be differentiated from *B. suis *by the presence of its own species specific SNP (Table 4).

## Conclusion

The data presented here have broad implications both in understanding the genetic diversity of the *Brucella *group and generating a robust taxonomic description, and in the development of potentially useful diagnostic tools. The scheme provides the basis for more extensive sampling of the *Brucella *group so that population diversity can be more fully estimated and isolates assigned to existing or new lineages. Clearly there are inconsistencies in the current taxonomy. We believe the data presented here will help generate discussion in this area and assist in resolving these issues. A strong argument could undoubtedly be made for reclassifying *B. canis *as an additional *B. suis *biovar, particularly given that the existing biovars already have distinct host specificities. *B. suis *biovar 5 could justifiably be removed from this group. The marine mammal isolates could be classified as a single species, but divided into subtypes corresponding to the host-specific STs described here or alternatively be classified as three distinct species on grounds of both (limited) genetic separation and apparent distinct host specificity. The existing classical species designations for *B. abortus*, *B. neotomae*, *B. ovis*, and *B. melitensis *appear genetically valid although the status of strain Tulya as an appropriate *B. abortus *biovar 3 reference strain needs investigation.

Clearly sequencing of the nine fragments described here is a potentially valuable tool for the identification of unknown *Brucella *isolates to classical species and/or biovar level the value of which can only increase as we add additional data to the extensive database already in place. Ongoing work is extending this database by both sequencing more targets to identify additional markers (particularly at the biovar level) and sequencing additional strains (particularly *B. abortus *and *B. melitensis*) in order to clarify, in conjunction with ongoing VNTR based studies [24], the relationships between genotype and biovar. Furthermore, the database provides a framework for placing any new or emerging *Brucella *groups in relation to current knowledge.

Finally, while sequencing the nine loci offers an excellent way of categorising *Brucella *isolates it is somewhat tedious and is not always a practical option. This study has identified a large number of well-defined species-specific markers, potentially useful for the development of diagnostic assays, that would avoid the need for such sequencing. The SNPs identified here have a number of advantages for use in such assays. As *Brucella *represents such a conserved group, and as most of the targets described here are housekeeping genes, SNPs are likely to have occurred only once in evolution. Equally, they are unlikely to mutate to new states or back to their ancestral state. Therefore, the phylogenetic framework presented here facilitates the confident selection of SNPs that define particular classical species, biovars or other groups. Assays based on these SNPs, that could offer a practical, robust and unambiguous alternative to biotyping, are under development in our laboratory and will be described elsewhere.

## Methods

### Bacterial isolates

A total of 160 isolates of *Brucella *were examined in this study. These represented all currently recognized classical species and biovars of *Brucella *(including all type strains and biovar reference strains) as well as an extensive collection of marine mammal isolates. The remaining sample was made up of field isolates from diverse hosts and geographic sources. Isolates were biotyped following standard procedure [42] although in some cases biovar designations reflect those provided by original strain suppliers. Templates for the PCR were prepared as described previously [24].

### PCR and sequencing

Nine distinct genome fragments were amplified by PCR using the primers shown in Table 1. PCR reaction mixes were prepared for each sample by mixing 5 μl FastStart 10× PCR Buffer with MgCl_2 _(Roche), 5 μl 2 mM dNTPs, 0.2 μl of each primer (at 100 pmol/μl), 0.25 μl of FastStart *Taq *DNA polymerase (Roche) and 39 μl of water. Routinely 0.5 μl of methanol extract or diluted genomic DNA was used as template. Cycling parameters were as follows: 94°C for 5 min. followed by 30 cycles of 94°C for 1 min., 53°C for 1 min. and 72°C for 1 min. and a polishing step of 72°C for 10 min. Products were separated by agarose gel electrophoresis to check for efficiency of amplification and to ensure that only a single product of the expected size (1) was present. PCR products were then purified by passage through QiaQuick PCR purification columns (Qiagen) and sequenced from either end using the same forward and reverse primers as used in initial PCR amplification. The Big Dye terminator cycle sequencing kit version 3.1 (Applied Biosystems) was used according to manufacturers instructions.

### Computer analysis of data

Sequence data was edited using the Lasergene package – manual editing was performed in the Editseq module while the SeqMan module was used to generate contigs from the forward and reverse sequences. Each distinct allele at each of the nine loci examined was given a distinct arbitrary numerical designation and each unique allelic pattern over all nine loci was identified as a sequence type (ST). Allelic profiles and sequence data were imported into the START package [43] to determine mean % GC content. The same package was used to calculate the average frequencies of synonymous substitutions per potential synonymous site (d_S_) and nonsynonymous substitutions per potential nonsynonymous site (d_N_) by the method of Nei and Gojobori [44] in order to test the degree of selection on a locus. The standardized I_A _(sI_A_), a measure that scales according to the number of loci analysed was calculated in the LIAN3.1 program [45,46]. LIAN3.1 was also used to test the null hypothesis of linkage equilibrium. A representative strain of each genotype (ST) was used for phylogenetic analysis. Sequences of the nine loci were concatenated to produce a 4,396 bp sequence for each genotype. Phylogenetic analysis was performed with the MEGA software, Version 3.1 [47]. Neighbour joining trees were constructed using the Jukes-Cantor model and the percentage bootstrap confidence levels of internal branches were calculated from 1000 resamplings of the original data. Split decomposition analysis [48] of allelic profile data was performed using a web-based version of the SplitsTree program [49].

### Sequence Accession Numbers

All sequences described in this study have been deposited in the EMBL database [EMBL: AM694191 through EMBL: AM695630].

## Authors' contributions

AMW conceived of and designed the study, carried out most of the experimental work, analyzed the data and drafted the manuscript.

LLP carried out biotyping, oversaw strain provision and provided expertise on conventional *Brucella *identification and typing.

APM provided intellectual input and expertise in relation to *Brucella *taxonomy.

All authors read and approved the final manuscript.

## Figures and Tables

**Table 1:** Oligonucleotide sequences used for the amplification and sequencing of nine genetic loci.

**Locus**	**Putative function**	**Primer sequences**	**Length**		**Location**^1^
*gap*	glyceraldehydes 3-phosphate dehydrogenase	5' YGCCAAGCGCGTCATCGT 3' 5' GCGGYTGGAGAAGCCCCA 3'	589 bp	AE017223	1685083–1685671
*aroA*	3-phosphoshikimate 1-carboxyvinyltransferase	5' GACCATCGACGTGCCGGG 3' 5' YCATCAKGCCCATGAATTC 3'	565 bp	AE017223	29974–30538
*glk*	glucokinase	5' TATGGAAMAGATCGGCGG 3' 5' GGGCCTTGTCCTCGAAGG 3'	475 bp	AE017224	988660–989134
*dnaK*	chaperone protein	5' CGTCTGGTCGAATATCTGG 3' 5' GCGTTTCAATGCCGAGCGA 3'	470 bp	AE017223	2066742–2067211
*gyrB*	DNA gyrase B subunit	5' ATGATTTCATCCGATCAGGT 3' 5' CTGTGCCGTTGCATTGTC 3'	469 bp	AE017223	142378–141910
*trpE*	anthranilate synthase	5' GCGCGCMTGGTATGGCG 3' 5' CKCSCCGCCATAGGCTTC 3'	486 bp	AE017223	1538194–1537709
*cobQ*	cobyric acid synthase	5' GCGGGTTTCAAATGCTTGGA 3' 5' GGCGTCAATCATGCCAGC 3'	422 bp	AE017223	1289341–1288920
*omp25*	25 kDa outer-membrane protein	5' ATGCGCACTCTTAAGTCTC 3' 5' GCCSAGGATGTTGTCCGT 3'	490 bp	AE017223	710041–710530
int-*hyp*	upstream and extreme 5' of hypothetical protein (BruAb1_1395)	5' CAACTACTCTGTTGACCCGA 3' 5' GCAGCATCATAGCGACGGA 3'	430 bp	AE017223	1372708–1372279

^1^Location in *B. abortus *9–941 genome sequence.

**Table 2:** Analysis of the nine loci examined in the *Brucella *strains sampled.

**Locus**	**Alleles**	**Polymorphic sites (%)**	**d_N_**	**d_S_**	**d_N_/d_S_**	**Mean % GC**
*gap*	6	7 (1.19)	0.0023	0.0105	0.217	58.33
*aroA*	7	7 (1.24)	0.0032	0.0058	0.559	61.68
*glk*	10	11 (2.32)	0.0051	0.0031	1.671	62.67
*dnaK*	7	6 (1.28)	0.0016	0.0103	0.156	60.71
*gyrB*	6	5 (1.07)	0.0027	0.0102	0.263	59.18
*trpE*	6	5 (1.03)	0.0033	0.0056	0.584	58.46
*cobQ*	7	8 (1.90)	0.0055	0.0129	0.425	59.15
*omp25*	10	12 (2.45)	0.0032	0.0161	0.196	59.08
int-*hyp*	5	6 (1.40)	-	-	-	55.75

d_N_= mean non-synonymous substitutions per non-synonymous site

d_S _= mean synonymous substitutions per synonymous site

**Table 3:** Origins of 160 *Brucella *strains examined in this study showing allelic profiles and ST designations.

	***gap***	***aroA***	***glk***	***dnaK***	***gyrB***	***trpE***	***cobQ***	***omp25***	***int-hyp***	**ST**	**Notes**	**Host**	**Source**
*B. abortus *544	2	1	1	2	1	3	1	1	1	1	Biovar 1 Reference		
*B. abortus *86/8/59	2	1	1	2	1	3	1	1	1	1	Biovar 2 Reference		
*B. abortus *292	2	1	1	2	1	3	1	1	1	1	Biovar 4 Reference		
*B. abortus *UK8/01	2	1	1	2	1	3	1	1	1	1	Biovar 1	Human	Eire
*B. abortus *I12	2	1	1	2	1	3	1	1	1	1	Biovar 1	Bovine	Northern Ireland
*B. abortus *UK25/01	2	1	1	2	1	3	1	1	1	1	Biovar 1	Human	Eire
*B. abortus *I103 (UK3/01)	2	1	1	2	1	3	1	1	1	1	Biovar 1	Bovine	Northern Ireland
*B. abortus *UK11/02	2	1	1	2	1	3	1	1	1	1	Biovar 1	Human	Eire
*B. abortus *63/59	2	1	1	2	1	3	1	1	1	1	NK	NK	Poland
*B. abortus *F6/04 04376	2	1	1	2	1	3	1	1	1	1	Biovar 1	Human	New Zealand
*B. abortus *R51/03	2	1	1	2	1	3	1	1	1	1	Biovar 1	Bovine	UK
*B. abortus *5/93	2	1	2	2	1	3	1	1	1	2	Biovar 3	Bovine	UK
*B. abortus *99/9971-159B	2	1	2	2	1	3	1	1	1	2	Biovar 7	NK	Mongolia
*B. abortus *biovar 5 B3196	2	1	2	2	1	3	1	1	1	2	Biovar 5 Reference		
*B. abortus *biovar 9 C68	2	1	2	2	1	3	1	1	1	2	Biovar 9 Reference		
*B. abortus *Sri Lanka 1	2	1	2	2	1	3	1	1	1	2	NK	Bovine	Sri Lanka
*B. abortus *03/4923-239	6	1	2	2	1	3	1	1	1	3	Biovar 7	Bovine	Turkey
*B. abortus *870	2	1	2	2	2	3	1	1	1	4	Biovar 6 Reference		
*B. abortus *S19	2	1	1	2	1	4	1	1	1	5	Vaccine Strain		
*B. abortus *RB51	2	1	1	2	1	4	1	1	1	5	Vaccine Strain		
*B. abortus *Tulya	5	7	10	7	6	3	7	1	1	6	Biovar 3 Reference		
*B. melitensis *biovar 1 16M	3	5	3	2	1	5	2	10	2	7	Biovar 1 Reference		
*B. melitensis *F3/02	3	5	3	2	1	5	2	10	2	7	Biovar 2	Human	Norway
*B. melitensis *B115	3	5	3	2	1	5	2	10	2	7	Rough Strain		
*B. melitensis *1BM1	3	5	3	2	1	5	2	10	2	7	Biovar 1	NK	Portugal
*B. melitensis *63/9	3	2	3	2	1	5	3	8	2	8	Biovar 2 Reference		
*B. melitensis *63/19	3	2	3	2	1	5	3	8	2	8	Biovar 2	Human	India
*B. melitensis *66/59	3	2	3	2	1	5	3	8	2	8	Biovar 3	Ovine	India
*B. melitensis *UK7/01	3	2	3	2	1	5	3	8	2	8	Biovar 1	Human	India
*B. melitensis *F8/01-155	3	2	3	2	1	5	3	8	2	8	Biovar 3	Bovine	Kosovo
*B. melitensis *65/155	3	2	3	2	1	5	3	8	2	8	Biovar 3	Ovine	Mongolia
*B. melitensis *UK23/01	3	2	3	2	1	5	3	8	2	8	Biovar 3	Human	Greece
*B. melitensis *F4/03-917	3	2	3	2	1	5	3	8	2	8	Biovar 1/3	Human	Greece
*B. melitensis *F3/05-373	3	2	3	2	1	5	3	8	2	8	Biovar 1	Ovine	Cyprus
*B. melitensis *UK10/05	3	2	3	2	1	5	3	8	2	8	Biovar 3	Human	UK
*B. melitensis *Ether	3	2	3	2	1	5	3	9	2	9	Biovar 3 Reference		
*B. melitensis *F12/01	3	2	3	5	1	5	2	10	2	10	Biovar 1	Ibex	UAE
*B. melitensis *80/82	3	2	3	2	1	5	3	10	2	11	Biovar 2	Human	Fmr Yugoslavia
*B. melitensis *UK31/99	3	2	3	2	1	5	3	10	2	11	Biovar 1	Human	UK
*B. melitensis *65/94	3	2	3	2	1	5	3	10	2	11	Biovar 3	Ovine	France
*B. melitensis *UK19/04	3	2	3	2	1	5	2	10	2	12	Biovar 1	Human	UK (Ethiopia)
*B. melitensis *R3903-60	3	2	3	2	1	5	2	10	2	12	Biovar 1	Livestock	Tanzania
*B. ovis *NCTC 63/290	1	3	9	2	1	3	4	3	1	13	*B. ovis *Type Strain		
*B. ovis *REO	1	3	9	2	1	3	4	3	1	13			
*B. ovis *79/60	1	3	9	2	1	3	4	3	1	13		Ovine	France
*B. ovis *63/96	1	3	9	2	1	3	4	3	1	13		Ovine	Argentina
*B. ovis *81/2	1	3	9	2	1	3	4	3	1	13		Ovine	Germany
*B. ovis *81/8	1	3	9	2	1	3	4	3	1	13		Ovine	Spain
*B. ovis *S92	1	3	9	2	1	3	4	3	1	13		Ovine	France
*B. ovis *80/125	1	3	9	2	1	3	4	3	1	13		Ovine	New Zealand
*B. ovis *79/69	1	3	9	2	1	3	4	3	1	13		Ovine	New Zealand
*B. ovis *79/160	1	3	9	2	1	3	4	3	1	13		Ovine	USA
*B. ovis *F10/D4/02	1	3	9	2	1	3	4	3	1	13		Ovine	New Zealand
*B. suis*1330	1	6	4	1	4	3	5	2	1	14	Biovar 1 Reference		
*B. suis *F7/03 BSI	1	6	4	1	4	3	5	2	1	14	Biovar 1	Porcine	Croatia
*B. suis *01-5744	1	6	4	1	4	3	5	2	1	14	Biovar 1	Porcine	Polynesia
*B. suis *64/24	1	6	4	1	4	3	5	2	1	14	Biovar 1	Porcine	USA
*B. suis *F1/04	1	6	4	1	4	3	5	2	1	14	Biovar 1	Human	Holland
*B. suis *F6/04 73/1616	1	6	4	1	4	3	5	2	1	14	Biovar 1	NK	New Zealand
*B. suis *63/176	1	6	4	1	4	3	5	2	1	14	Biovar 1	Human	Tunisia
*B. suis *92/29	1	6	4	1	4	3	5	2	1	14	Biovar 1	Human	Mexico
*B. suis *RT1	1	6	4	1	4	3	5	2	1	14	Biovar 1/3	Equine	Croatia
*B. suis *RT2	1	6	4	1	4	3	5	2	1	14	Biovar 1	Human	NK
*B. suis *RT3	1	6	4	1	4	3	5	2	1	14	Biovar 1/3	Porcine	Croatia
*B. suis *Thomsen	1	2	7	1	3	3	5	2	3	15	Biovar 2 Reference		
*B. suis *F12/02	1	2	7	1	3	3	5	2	3	15	Biovar 2	Hare	Denmark
*B. suis *F13/02	1	2	7	1	3	3	5	2	3	15	Biovar 2	Hare	Denmark
*B. suis *74/12	1	2	7	1	3	3	5	2	3	15	Biovar 2	NK	Denmark
*B. suis *F5/03-2	1	2	7	1	3	3	5	2	3	15	Biovar 2	Porcine	Portugal
*B. suis *79/194	1	2	7	1	3	3	5	2	3	15	Biovar 2	Hare	Czechslovakia
*B. suis *23	1	2	7	1	3	3	5	2	3	15	Biovar 2	NK	Portugal
*B. suis *31	1	2	7	1	3	3	5	2	3	15	Biovar 2	NK	Portugal
*B. suis *32	1	2	7	1	3	3	5	2	3	15	Biovar 2	NK	Portugal
*B. suis *RT4	1	2	7	1	3	3	5	2	3	15	Biovar 2	Hare	France
*B. suis *RT5	1	2	7	1	3	3	5	2	3	15	Biovar 2	Hare	France
*B. suis *RT6	1	2	7	1	3	3	5	2	3	15	Biovar 2	Porcine	France
*B. suis *RT7	1	2	7	1	3	3	5	2	3	15	Biovar 2	Porcine	France
*B. suis *RT8	1	2	7	1	3	3	5	2	3	15	Biovar 2	Hare	France
*B. suis *RT9	1	2	7	1	3	3	5	2	3	15	Biovar 2	Porcine	France
*B. suis *RT10	1	2	7	1	3	3	5	2	3	15	Biovar 2	Porcine	Portugal
*B. suis *RT11	1	2	7	1	3	3	5	2	3	15	Biovar 2	Hare	France
*B. suis *RT12	1	2	7	1	3	3	5	2	3	15	Biovar 2	Bovine	France
*B. suis *RT13	1	2	7	1	3	3	5	2	3	15	Biovar 2	Porcine	Portugal
*B. suis *RT14	1	2	7	1	3	3	5	2	3	15	Biovar 2	Wild Boar	France
*B. suis *RT15	1	2	7	1	3	3	5	2	3	15	Biovar 2	Porcine	France
*B. suis *RT16	1	2	7	1	3	3	5	2	3	15	Biovar 2	Hare	France
*B. suis *RT17	1	2	7	1	3	3	5	2	3	15	Biovar 2	Wild Boar	Italy
*B. suis *RT18	1	2	7	1	3	3	5	2	3	15	Biovar 2	Wild Boar	Switzerland
*B. suis *74/11	1	2	7	1	3	3	5	2	3	15	Atypical	NK	Denmark
*B. suis *92/63	1	2	7	1	3	3	5	2	3	15	Atypical	Hare	Slovenia
*B. suis *94/11	4	2	7	1	3	3	5	2	3	16	Atypical Biovar 2	Porcine	Bulgaria
*B. suis *RT19	4	2	7	1	3	3	5	2	3	16	Biovar 2	Porcine	France
*B. suis *RT20	4	2	7	1	3	3	5	2	3	16	Biovar 2	Wild Boar	Germany
*B. suis *RT21	4	2	7	1	3	3	5	2	3	16	Biovar 2	Porcine	Croatia
*B. suis *686	1	6	4	1	5	3	5	2	4	17	Biovar 3 Reference		
*B. suis *63/34	1	6	4	1	5	3	5	2	4	17	Biovar 3	NK	USA
*B. suis *40	1	6	4	1	5	3	5	2	4	17	Biovar 4 Reference		
*B. suis *63/202	1	6	4	1	5	3	5	2	4	17	Biovar 4	Reindeer	Fmr USSR
*B. suis *63/252	1	6	4	1	5	3	5	2	4	17	Biovar 4	Caribou	USA (Alaska)
*B. suis *79/30	1	6	4	1	5	3	5	2	4	17	Biovar 4	Human	Finland
*B. suis *63/219	1	6	4	1	5	3	5	2	4	17	Biovar 4	Reindeer	Fmr USSR
*B. suis *63/198	1	6	4	1	5	3	5	2	5	18	Biovar 4	Reindeer	Fmr USSR
*B. suis *513	1	2	4	6	1	3	5	2	1	19	Biovar 5 Reference		
*B. canis *RM6/66	1	6	4	1	5	3	5	6	4	20	*B. canis *Type Strain		
*B. canis *UK10/02	1	6	4	1	5	3	5	6	4	20		Canine	UK
*B. canis *79/85	1	6	4	1	5	3	5	6	4	20		Canine	Peru
*B. canis *79/92	1	6	4	1	5	3	5	6	4	20		Canine	Germany
*B. canis *79/139	1	6	4	1	5	3	5	6	4	20		Canine	USA
*B. canis *F7/02	1	6	4	1	5	3	5	5	4	21		Canine	Germany
*B. canis *F7/05A	1	6	4	1	5	3	5	5	4	21		Canine	South Africa
*B. canis *F7/05B	1	6	4	1	5	3	5	5	4	21		Canine	South Africa
*B. neotomae *5K33	1	2	5	2	1	6	5	4	1	22	*B. neotomae *Type Strain		
*B. neotomae *65/196	1	2	5	2	1	6	5	4	1	22		Desert Wood Rat	USA
*B. neotomae *65/197	1	2	5	2	1	6	5	4	1	22		Desert Wood Rat	USA
*Brucella *sp. 36/94	1	4	8	4	1	2	5	2	1	23		Harbour Porpoise	UK
*Brucella *sp. 52/94	1	4	8	4	1	2	5	2	1	23		Harbour Porpoise	UK
*Brucella *sp. UK15/98	1	4	8	4	1	2	5	2	1	23		Harbour Porpoise	UK
*Brucella *sp. UK35/99	1	4	8	4	1	2	5	2	1	23		Harbour Porpoise	UK
*Brucella *sp. 14/95	1	4	8	4	1	2	5	2	1	23		Common Seal	UK
*Brucella *sp. 2/96	1	4	8	4	1	2	5	2	1	23		Harbour Porpoise	UK
*Brucella *sp. UK1/97	1	4	8	4	1	2	5	2	1	23		Harbour Porpoise	UK
*Brucella *sp. UK3/97	1	4	8	4	1	2	5	2	1	23		Common Dolphin	UK
*Brucella *sp. UK5/97	1	4	8	4	1	2	5	2	1	23		Whitesided Dolphin	UK
*Brucella *sp. UK10/00	1	4	8	4	1	2	5	2	1	23		Harbour Porpoise	UK
*Brucella *sp. UK4/01	1	4	8	4	1	2	5	2	1	23		Harbour Porpoise	UK
*Brucella *sp. UK10/01	1	4	8	4	1	2	5	2	1	23		Harbour Porpoise	UK
*Brucella *sp. UK15/02	1	4	8	4	1	2	5	2	1	23		Harbour Porpoise	UK
*Brucella *sp. F23/97	1	4	8	4	1	2	5	2	1	23		Dolphin	France
*Brucella *sp. F96/2	1	4	8	4	1	2	5	2	1	23		Harbour Porpoise	Germany
*Brucella *sp. VLA04.67	1	4	8	4	1	2	5	2	1	23		Seal	UK
*Brucella *sp. VLA04.72	1	4	8	4	1	2	5	2	1	23		Porpoise	UK
*Brucella *sp. VLA04/105	1	4	8	4	1	2	5	2	1	23		Porpoise	UK
*Brucella *sp. VLA04/06	1	4	8	4	1	2	5	2	1	23		Harbour Porpoise	UK
*Brucella *sp. UK31/04	1	4	8	4	1	2	5	2	1	23		Porpoise	UK
*Brucella *sp. 39/94	1	2	6	2	1	2	5	2	1	24		Common Seal	UK
*Brucella *sp. 44/94	1	2	6	2	1	2	5	2	1	24		Common Seal	UK
*Brucella *sp. UK24/00 (M192)	1	2	6	2	1	2	5	2	1	24		Minke Whale	UK
*Brucella *sp. 55/94	1	2	4	2	1	2	5	2	1	25		Otter	UK
*Brucella *sp. 4/96	1	2	4	2	1	2	5	2	1	25		Common Seal	UK
*Brucella *sp. 61/94	1	2	4	2	1	2	5	2	1	25		Grey Seal	UK
*Brucella *sp. UK13/99	1	2	4	2	1	2	5	2	1	25		Common Seal	UK
*Brucella *sp. UK40/99	1	2	4	2	1	2	5	2	1	25		Grey Seal	UK
*Brucella *sp. UK5/01	1	2	4	2	1	2	5	2	1	25		Common Seal	UK
*Brucella *sp. F6/99	1	2	4	2	1	2	5	2	1	25		Harbour Seal	USA
*Brucella *sp. F8/99	1	2	4	2	1	2	5	2	1	25		Harbour Seal	USA
*Brucella *sp. F9/99	1	2	4	2	1	2	5	2	1	25		Harbour Seal	USA
*Brucella *sp. F10/99	1	2	4	2	1	2	5	2	1	25		Harbour Seal	USA
*Brucella *sp. F7/99	1	2	4	2	1	2	5	2	1	25		Bottlenosed Dolphin	USA
*Brucella *sp. UK28/03-13840	1	2	4	2	1	2	5	2	1	25		Seal	UK
*Brucella *sp. 59/94	1	2	4	2	1	2	6	7	1	26		Striped Dolphin	UK
*Brucella *sp. 5/95	1	2	4	2	1	2	6	7	1	26		Striped Dolphin	UK
*Brucella *sp. UK43/99	1	2	4	2	1	2	6	7	1	26		Striped Dolphin	UK
*Brucella *sp. UK1/2000	1	2	4	2	1	2	6	7	1	26		Striped Dolphin	UK
*Brucella *sp. UK2/2000	1	2	4	2	1	2	6	7	1	26		Striped Dolphin	UK
*Brucella *sp. 14/94	1	2	4	2	1	2	6	7	1	26		Common Dolphin	UK
*Brucella *sp. UK3/05	1	2	4	2	1	2	6	7	1	26		Striped Dolphin	UK
*Brucella *sp. VLA05/4	1	2	4	2	1	2	6	7	1	26		Bottlenosed Dolphin	UK
*Brucella *sp. VLA05/8	1	2	4	2	1	2	6	7	1	26		Common Dolphin	UK
*Brucella *sp. F5/99	1	2	4	3	1	1	5	2	1	27		Bottlenosed Dolphin	USA
*Brucella *sp. F5/02	1	2	4	3	1	1	5	2	1	27		Human	New Zealand

NK = Not Known

**Table 4:** Diversity within *Brucella *species and identification of species-specific polymorphisms.

	**Number of STs**	**Number of intraspecies polymorphisms**	**Species specific polymorphisms**
***B. abortus***	6	14 (4 without ST6 Tulya)	2 (*aroA*-647; *omp25*-3627)
***B. melitensis***	6	5	11 (*gap*-163; *gap *– 323; *glk *– 1227; *glk *– 1403; *trpE*-3048; *cobQ*-3387; *omp25*-3499; *omp25 *– 3828; int-*hyp*- 4064; int-*hyp *– 4260; int-*hyp*-4356)
***B. ovis***	1	0	6 (*aroA*-1085; glk-1557; *glk*-1578; *cobQ*-3363; *omp25*-3745; *omp25*-3864)
***B. suis***	6	12 (9 without ST19 biovar 5)	0 (none specific for all *B. suis *though there are polymorphisms specific for biovars 2 and 5)
***B. canis***	2	1	1 (*omp25*-3715)
***B. neotomae***	1	0	3 (*trpE*-2798;*omp25 *– 3498; *omp25*-3845)
**Marine mammal *Brucella***	5	8	1 (*trpE*-2858)

**Table 5:** Multilocus linkage disequilibrium analysis of the 160 *Brucella *isolates examined.

**Group (n)**	**V**_**e**_^**a**^	**V**_**o**_^**a**^	**sI**_**A**_^**b**^	***P***^**b**^
Total isolates (160)	2.022	5.719	0.2286	0.001
Total STs (27)	1.825	4.679	0.1954	0.001
*B. abortus *(21)	0.958	2.913	0.2549	0.001
*B. abortus *STs (6)	1.564	5.067	0.2798	0.007
*B. melitensis *(21)	0.799	1.302	0.0787	0.001
*B. melitensis *STs (6)	0.924	0.695	-0.0310	0.808
*B. suis *(50)	1.154	3.646	0.2699	0.001
*B. suis *STs (6)	1.164	1.981	0.0876	0.054
Marine mammal (46)	1.257	3.171	0.1903	0.001
Marine mammal STs (5)	1.380	1.333	-0.0042	0.557

^a ^V_e_is the expected variance if the alleles present at different loci in a strain are independent (i.e. no linkage disequilibrium), V_o _is the observed variance.

^b ^Standardised I_A _[26] calculated using LIAN3.1. *P *= probability of observing a V_o_/V_e _ratio equal or more extreme to that found in the original data based on 1,000 Monte Carlo randomisations.
